# Ultraviolet Radiation-Induced Production of Nitric Oxide:A multi-cell and multi-donor analysis

**DOI:** 10.1038/s41598-017-11567-5

**Published:** 2017-09-11

**Authors:** Graham Holliman, Donna Lowe, Howard Cohen, Sarah Felton, Ken Raj

**Affiliations:** 1 Radiation Effects Department, Centre for Radiation, Chemical and Environmental Hazards,Public Health England (PHE), Chilton, Oxfordshire, OX11 0RQ United Kingdom; 2 Elizabeth House, 515 Limpsfield Road, Warlingham, Surrey CR6 9LF United Kingdom; 3 Oxford University Hospitals NHS Foundation Trust, Old Road, Oxford, OX3 7LJ United Kingdom

## Abstract

Increasing evidence regarding positive effects of exposure to sunlight has led to suggestions that current advice may be overly weighted in favour of avoidance. UV-A has been reported to lower blood pressure, possibly through nitric oxide (NO) production in skin. Here, we set out to investigate effects of UV-A and solar-simulated radiation on the potential source of dermal NO, the effective doses and wavelengths, the responsiveness of different human skin cells, the magnitude of inter-individual differences and the potential influence of age. We utilised isogenic keratinocytes, microvascular endothelial cells, melanocytes and fibroblasts isolated from 36 human skins ranging from neonates to 86 years old. We show that keratinocytes and microvascular endothelial cells show greatest NO release following biologically relevant doses of UV-A. This was consistent across multiple neonatal donors and the effect is maintained in adult keratinocytes. Our observations are consistent with a bi-phasic mechanism by which UV-A can trigger vasodilatory effects. Analyses of NO-production spectra adds further evidence that nitrites in skin cells are the source of UV-mediated NO release. These potentially positive effects of ultraviolet radiation lend support for objective assessment of environmental influence on human health and the idea of “healthy sun exposure”.

## Introduction

In spite of the well-known detrimental effects of over-exposure to sunlight and ultraviolet (UV) radiation, devising public health advice in this regard is not straightforward as exposure to sunlight also brings benefits to health. The most studied and well-characterised benefit is the UV-B-mediated synthesis of vitamin D^1, 2^. Clearly, a balance between exposure and avoidance must be struck and this challenging aim must be constantly re-assessed in the face of new evidence of sunlight effects, whether detrimental or beneficial. Lately, reports on the potential of sunlight to reduce blood pressure have begun to emerge^3–8^. If true and significant, this beneficial effect must be included in the balance upon which public health advice is based.

Nitric oxide (NO) is a potent vasodilator. It is generated primarily by the enzyme nitric oxide synthase (NOS), of which there are three identified subtypes –NOS1/nNOS, NOS2/iNOS and NOS3/eNOS. These oxidise L-arginine to produce NO^9^, which can subsequently be oxidised to nitrites and nitrates, among other species^10^. The initial assumption that nitrites and nitrates were merely by-products of NO metabolism without any function has been disproven by many reports demonstrating them to be important bio-active molecules^10, 11^. Besides the suggestion of activity in their own right, various mechanisms by which NO can be liberated from nitrates and nitrites have been reported^12–14^, including the action of sunlight^5, 15, 16^. These observations suggest that they may represent an important reservoir from which NO could be generated through mechanisms that are independent of NOS enzyme activity^12, 17–19^. The fact that the skin contains nitrite at levels approximately 25 times higher than that found in human plasma lends credence to this notion^20^.

The generation of NO in human keratinocytes was first observed over twenty years ago, as a response to TNFα or IFNα treatment^21^. Since then many other cytokines have been found to also induce keratinocytes to produce NO. This response is believed to be part of the skin’s defence and repair mechanism, as NO is involved in inflammation, microbicidal activity, apoptosis and wound healing^20, 22, 23^. Perhaps the most interesting mechanism of dermal NO production is via exposure to the UV content of sunlight. UV radiation has varied effects on the skin, including erythema, immunosuppression, DNA damage and vitamin D synthesis, as well as activating a large variety of bio-active molecules, which have been the subject of much investigation^23, 24^. In many of these UV-induced processes NO is reportedly involved, with UV either activating or increasing expression of NOS enzymes. It has been demonstrated that exposure to UV can result in an increase in the level of iNOS in the skin 8–10 hours post-exposure^17, 25^. Although this is one way by which UV can increase NO in cells, it is not the sole means; studies have reported NO released so rapidly following exposure that this cannot be accounted for by increased synthesis of NOS enzymes^5^. Importantly the amount of NO released immediately following exposure was sufficient to reduce blood pressure^8, 16, 20^. This strongly suggests that UV can induce a non-enzymatic mechanism to augment NO at physiologically relevant levels^20^.

It is felt that the UV-A portion of the solar spectrum, which ranges from 315 nm to 400 nm, is responsible for this rapid NO production. Furthermore, whole-body UV-A irradiation was reportedly able to cause a reduction in blood pressure in humans during exposure, and for some time after^5, 8^. The UV spectrum is divided into UV-A (400–315 nm), UV-B (315–280 nm) and UV-C (280–100 nm). UV-C is the most damaging to DNA, but in terms of public exposure from the environment is irrelevant as it is filtered out by ozone and other components of the atmosphere and is not present in the solar spectrum at ground level. UV-B on the other hand which penetrates into the epidermis of the skin, is capable of inducing significant damage to DNA, while UV-A, which penetrates even deeper into the dermal layer, is thought to be far less directly damaging to DNA. If release of NO is mediated purely by UV-A, with little or no contribution from UV-B, it could increase the appeal of using UV-A as a natural vasodilator. While the bulk of direct DNA damage is attributed to UV-B, UV-A is still responsible for undesirable effects, including oxidative damage, skin photo-ageing and immunosuppressive effects^26–28^. As mentioned, UV-A’s ability to penetrate further into the skin than UV-B also means that any negative, or positive, interactions in the skin may occur at greater depths. As such, UV-A cannot be considered intrinsically ‘safe’, but its safety must be considered on balance of the risk to benefit ratio.

A recent epidemiological report demonstrated a lower cumulative incidence of myocardial infarction (MI) in individuals with either non-melanoma or malignant melanoma skin cancer compared to individuals without^29^. This assumes the prevalence of skin cancer as a surrogate marker for lifetime exposure to UV radiation. While this intriguing report is of substantial interest, there are many potential confounding factors and there additionally exists a gap between the studies demonstrating acute effect of UV irradiation lowering blood pressure and the lifetime exposure to UV and lower MI risk. In spite of these promising observations, there remain many questions which must be very carefully addressed before responsible and evidence-based advice can be formulated. These include whether exposure to UV radiation always has a blood pressure-lowering effect, or if the effect of acute exposure is lost when the exposure becomes chronic or repeated? In addition to these questions, there are uncertainties as to the identity of cells in the skin that are responsible for UV-induced NO production, the magnitude of UV-induced NO increase between individuals, the longevity of the effect, any consequences of cumulative exposures and the long-term effects of protracted UV-A exposure. There is also the intriguing question of how NO, a very short lived compound, believed to be produced in the upper layers of the skin, can rapidly induce dilation of blood vessels which reside in the dermis, to the extent that systemic blood pressure is reduced.

As part of our effort to address some of these questions we began by characterising the different effects of UV-A and UV-B in terms of their effects on DNA, devising a reliable and sensitive assay to ascertain the ability of UV-A or UV-B to activate NO production, investigate the response of various skin cell types with regards to their ability to generate NO in response to UV, ascertaining the reservoir source of UV-A-inducible NO and to investigate the magnitude of inter-individual differences in regards to NO production by UV.

## Results

### DNA damage potential

**Figure 1:**
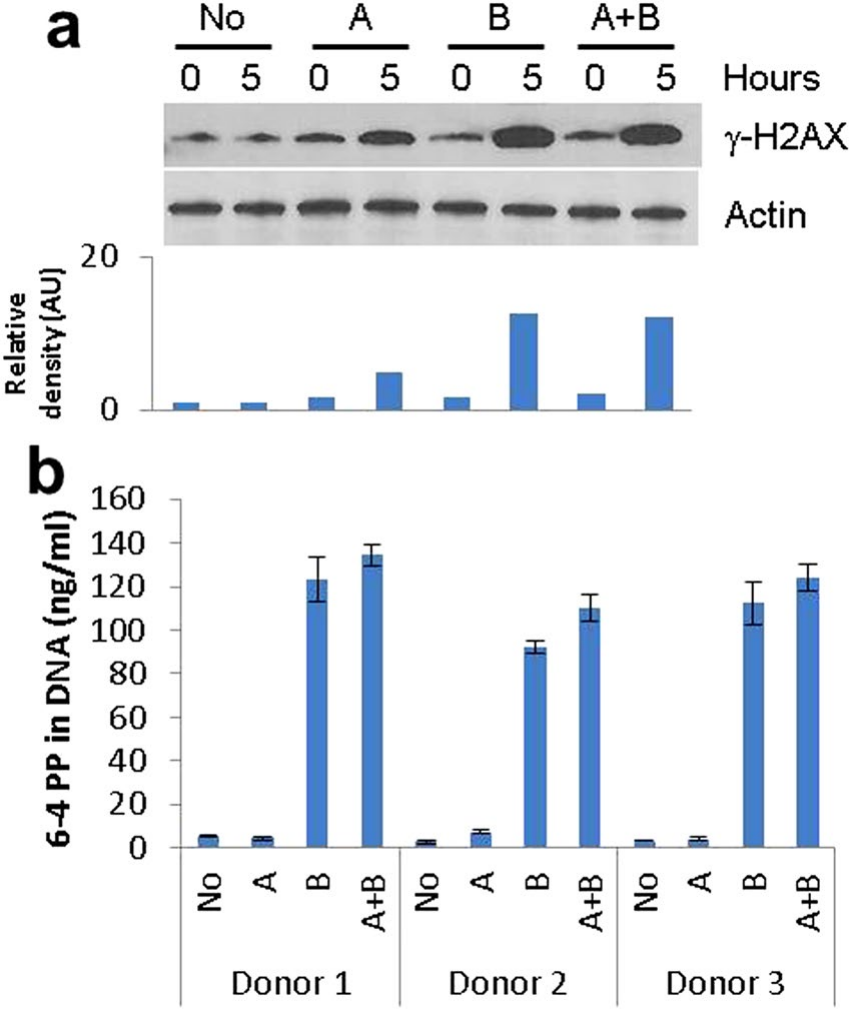
Assessment of UV-A and UV-B’s relative ability to damage DNA. **(a)** γ-H2AX western blot of lysates from primary FSKs irradiated with No UV (No), 9 J/cm^2^ UV-A (A), 0.09 J/cm^2^ UV-B (B) or both 9 J/cm^2^ UV-A and 0.09 J/cm^2^ UV-B (A + B) harvested at either 0 or 5 hours (0 or 5) post-irradiation. Densitometry of γ-H2AX levels, normalised to actin, is given showing the relative intensity of the bands. (Images shown here are cropped from larger scanned images. Complete gel scans are included as Supplementary Figure 1). **(b)** 6-4PP formation in DNA from primary FSKs isolated from three donor skins immediately following exposure to No UV (No), 9 J/cm^2^ UV-A (A), 0.09 J/cm^2^ UV-B (B) or both 9 J/cm^2^ UV-A and 0.09 J/cm^2^ UV-B (A + B). (Error bars are standard deviations of duplicate samples).

The reported ability of UV-A to reduce blood pressure through NO generation in the skin is highly relevant especially in regards to health advice on sun exposure and eventually, the possible use of UV-A as an aid in modulating blood pressure. In spite of this attractive potential, UV radiation is still a proven carcinogen. As such, it is important to ascertain the relative effects of these different UV radiations (A and B) in terms of DNA damage. Previous studies using various cell types reported UV-A and UV-B to have very different damaging effects on cells. To determine first-hand how these UV radiations affect primary human skin cells, we exposed primary human foreskin keratinocytes (FSKs) to UV-A or UV-B with 9 J/cm^2^ and 0.09 J/cm^2^ respectively. These doses were selected on the basis that sunlight at noon on a clear summer’s day in the UK would give a dose of 9 J/cm^2^ of UV-A in approximately 35 minutes^30, 31^. To limit inaccuracies that can arise when measurement of vastly different quantities are made within a single assay, UV-B was delivered at a considerably lower dose as it is estimated to be approximately a hundred times more damaging than UV-A^32, 33^. Following irradiation, total cell proteins were harvested both immediately post-irradiation and after a further 5 hours of culture. As can be seen in Fig. 1a, UV-B readily induced phosphorylation of H2AX, which is indicative of DNA double strand breaks, above that of un-irradiated cells, while UV-A had a more limited effect in spite of it being delivered at a dose a hundred times greater.

Since the predominant type of damage to DNA caused by UV is the formation of pyrimidine dimers, we tested for these with primary FSKs isolated from three donor foreskins, in order to avoid anomalous results that could arise from using cells from a single donor. Immediately following exposures to UV-A, UV-B or both UV-A and UV-B, DNA was collected, and the amounts of 6,4 pyrimidine-pyrimidone (6-4PP) photoproducts were measured using an ELISA (Fig. 1b). Once again, UV-B exhibited significantly greater damage to DNA in the form of pyrimidine dimers, in spite of the fact that the delivered dose was a hundred times smaller than that of UV-A.

### Nitric oxide production in human foreskin cells

**Figure 2:**
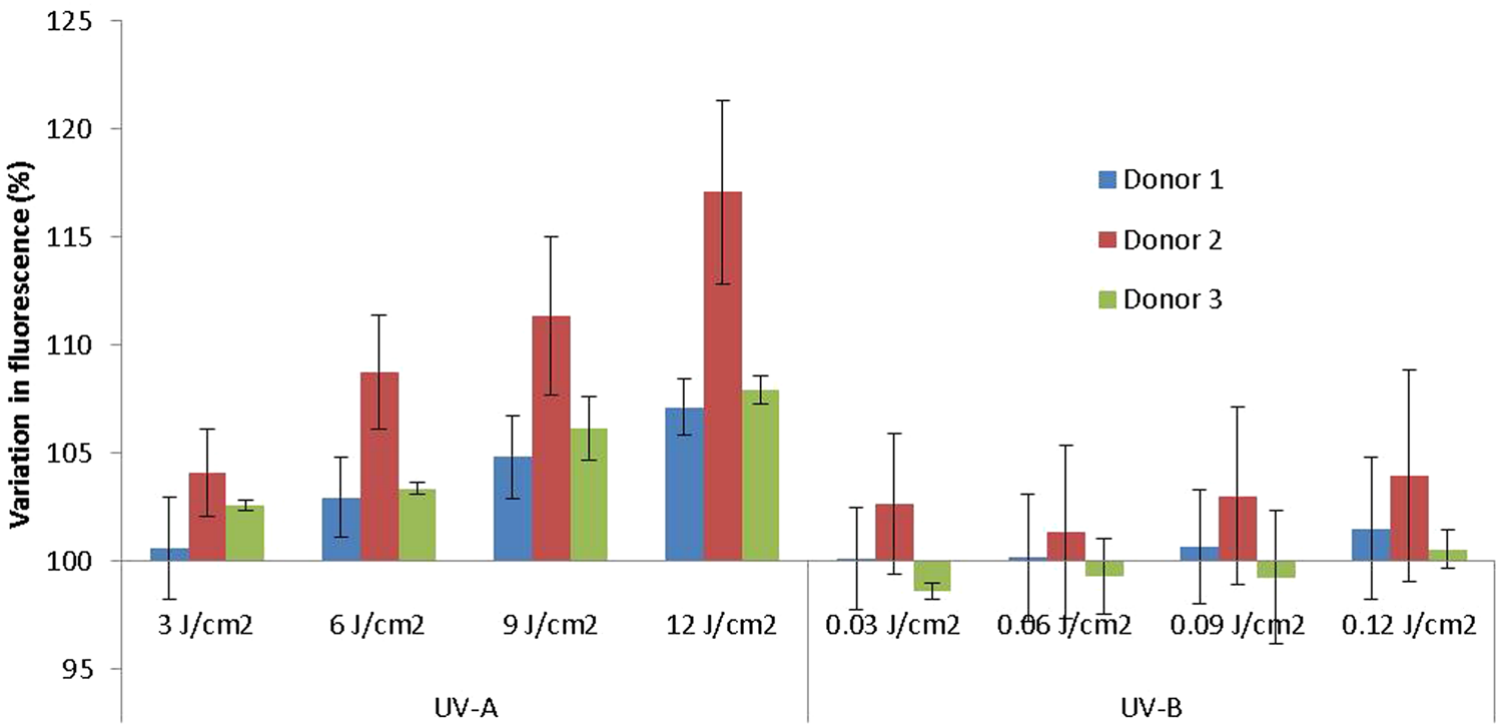
Comparison of NO generation with UV-A and UV-B in FSKs from three donors. Primary FSKs were irradiated with either 3, 6, 9 or 12 J/cm^2^ UV-A or 0.03, 0.06, 0.09 or 0.12 J/cm^2^ UV-B. Increase of NO levels were measured by flow cytometry of DAF-FM fluorescence in FSKs from three donor foreskins. Results are expressed as % change in fluorescence relative to unexposed control. (Error bars are standard deviations of triplicate readings. Donor numbers differ from those used in other figures).

Having established the relatively small effect of UV-A on DNA damage, we proceeded to test the ability of UV-A to induce NO production in cells. To this end, we used DAF-FM DA, which can be readily taken into cells and, once inside, is cleaved by cellular esterases to become DAF-FM, which is a dye that interacts rapidly and irreversibly with NO, becoming fluorescent, allowing measurement of relative NO levels. Beginning with FSKs from another three donors (different from those in Fig. 1b), we subjected these cells to 3, 6, 9 or 12 J/cm^2^ of UV-A or 0.03, 0.06, 0.09 or 0.12 J/cm^2^ of UV-B. For all three donors, UV-A-mediated NO rise showed a clear dose-dependent relationship with increasing UV-A dose, confirming the ability of UV-A to do this at doses that are within environmental parameters (Fig. 2). The response to UV-B does show a general trend of increasing NO level with UV-B dose, however, this is much lower than the level seen with UV-A.

**Figure 3:**
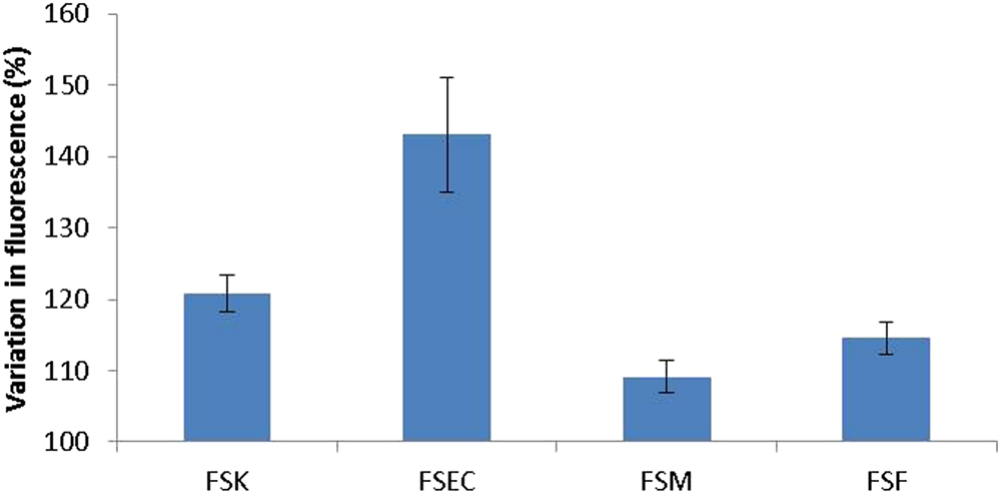
Comparison of NO generation in four cell types derived from a single donor foreskin. Primary FSKs, FSECs, FSMs and FSFs were irradiated with 9 J/cm^2^ UV-A following incubation with DAF-FM DA dye. Increase of NO levels were measured by flow cytometry. Results are expressed as % change in fluorescence relative to unexposed control. (Error bars are standard deviations of triplicate readings).

It is appreciated that NO is a particularly reactive radical with a very short half-life in the biological environment. As such, it is not immediately obvious how its production and release from skin cells such as keratinocytes, as was reported^16^, can have a dilatory effect on smooth muscle cells located around blood vessels which lie a considerable distance from the epidermis. To begin addressing this, we set out to ascertain how various cell types within the skin respond to UV-A by producing NO. We employed methods that we have developed to isolate keratinocytes, fibroblasts, melanocytes and microvascular endothelial cells from the same piece of foreskin. The resulting cells are isogenic and can be used in experiments to determine genuine differences between cell types without the uncertainty of potential contribution of genetic differences. The responses of these four isogenic cell types to UV-A irradiation were tested using DAF-FM DA. The results revealed that FSKs and microvascular endothelial cells (FSECs) generated the greatest relative increases in NO while melanocytes (FSMs) and fibroblasts (FSFs) show smaller increases (Fig. 3). This is the first demonstration of the ability of non-keratinocyte skin cells, especially microvascular endothelial cells, to respond to UV-A in this manner.

**Figure 4:**
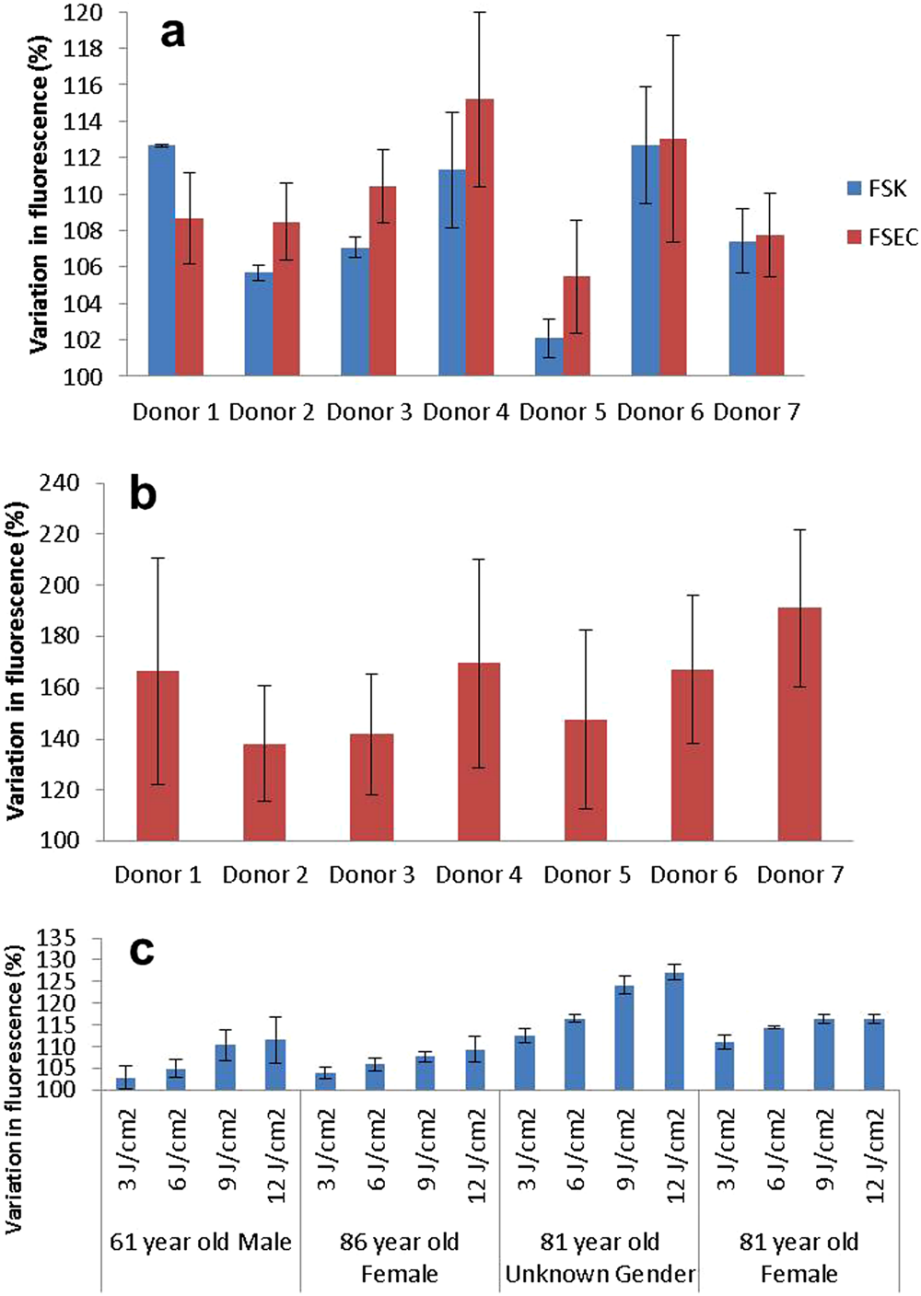
Comparison of NO generation in FSK and FSEC matched pairs from seven donors. (**a**) Primary FSKs and FSECs isolated from the same donors were irradiated with 9 J/cm^2^ UV-A following incubation with DAF-FM DA dye. Increase of NO levels were measured by flow cytometry. Results are expressed as % change in fluorescence relative to unexposed control. (Error bars are standard deviations of triplicate readings. Donor numbers differ from those used in other figures). (**b**) Primary FSECs irradiated with 9 J/cm^2^ UV from a solar simulator. Results are expressed as % change in fluorescence relative to unexposed control. (Error bars are standard deviations of triplicate readings. Donor numbers differ from those used in other figures). (**c**) Primary adult keratinocytes from 4 adult donors (61 year old male; 81 and 86 year old females; and 81 year old unknown gender), exposed to UV-A at 3, 6, 9 and 12 J/cm^2^ with DAF-FM DA used to measure NO production. Results are expressed as % change in fluorescence relative to unexposed control. (Error bars are standard deviations of triplicate readings).

To further investigate this interesting observation and ensure that this is not due to an anomaly of a single donor, we isolated FSECs and FSKs from 7 other donors, irradiated them with UV-A and measured NO production. The results show that FSECs are indeed as responsive, if not more responsive, to UV-A in this regard than FSKs (Fig. 4a). The significance of the FSECs’ responsiveness to UV-A is very interesting and will be discussed further below. To ascertain if this phenomenon also occurs in response to an environmentally relevant light spectrum, FSECs from seven other donors were irradiated not with just UV-A but in a solar simulator with 9 J/cm^2^ instead. The NO produced was measured using DAF-FM DA (Fig. 4b). Consistent increases in NO level were seen, with seemingly even greater increases than with UV-A alone. This is of much interest, and worthy of further study, as it indicates the contributing role of light at other wavelengths (either UV-B, visible or infra-red wavelengths) in generating NO, either from the same or different reservoirs. Most importantly, these experiments demonstrate that NO production is not attenuated by light spectrum that is akin to sunlight; instead the effect is even more pronounced.

### NO production in adult skin cells

To ensure that the observed UV-mediated NO production in isolated foreskin cells was not a phenomenon particular to neonatal tissue, we isolated keratinocytes from samples of adult skin from four donors (a 61 year old male; 81 and 86 year old females; and an 81 year old of unknown gender). Exposing the keratinocytes to four different doses of UV-A demonstrated dose-dependent NO production visible in all four donors (Fig. 4c).

### Inter-individual variation in NO production

**Figure 5:**
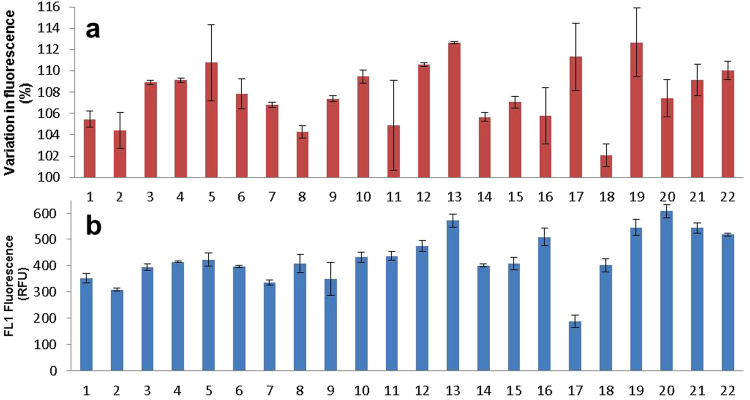
Comparison of response to UV-A irradiation in FSKs from 22 different donors. (**a**) Percentage increase of NO levels as measured by flow cytometry of DAF-FM DA fluorescence in 22 different donor primary FSK samples following exposure to 9 J/cm^2^ UV-A. Results are expressed as % change in fluorescence relative to unexposed control. (Error bars are standard deviations of duplicate samples). (**b**) Comparison of relative basal levels of NO as assessed by DAF-FM DA fluorescence in un-irradiated primary FSKs from 22 donors. (Error bars are standard deviations of duplicate samples).

To ascertain whether the potentially beneficial effect of UV-A through the generation of NO is affected by inter-individual variations, primary FSKs from a further 22 different donors were isolated and variation in NO was tested following UV-A irradiation. When plotted as percentage change in fluorescence between UV-exposed and unexposed cells, variations between donors are apparent (Fig. 5a). This is very interesting and deserves further investigation to ascertain what affects responsiveness to UV-A. Nonetheless, for the current purpose, the results show that a consistent increase in NO production is seen, with an average of approximately 7% across the 22 donors.

While these experiments were designed to ascertain the magnitude of inter-individual differences in regards to UV-A-induced NO production, we were surprised to observe differences between donors in the quantity of NO already present, or produced, in their cells without irradiation (Fig. 5b). Indeed, some of these differences were even greater than the UV-induced NO between the donors. Interestingly, the magnitude of rise in UV-induced NO seemed unrelated to the initial NO level in the un-irradiated donor cells. This would suggest that the source of UV-A-induced NO is likely to be different from that generated endogenously, independently of UV.

### Production of NO from nitrite and nitrate

**Figure 6:**
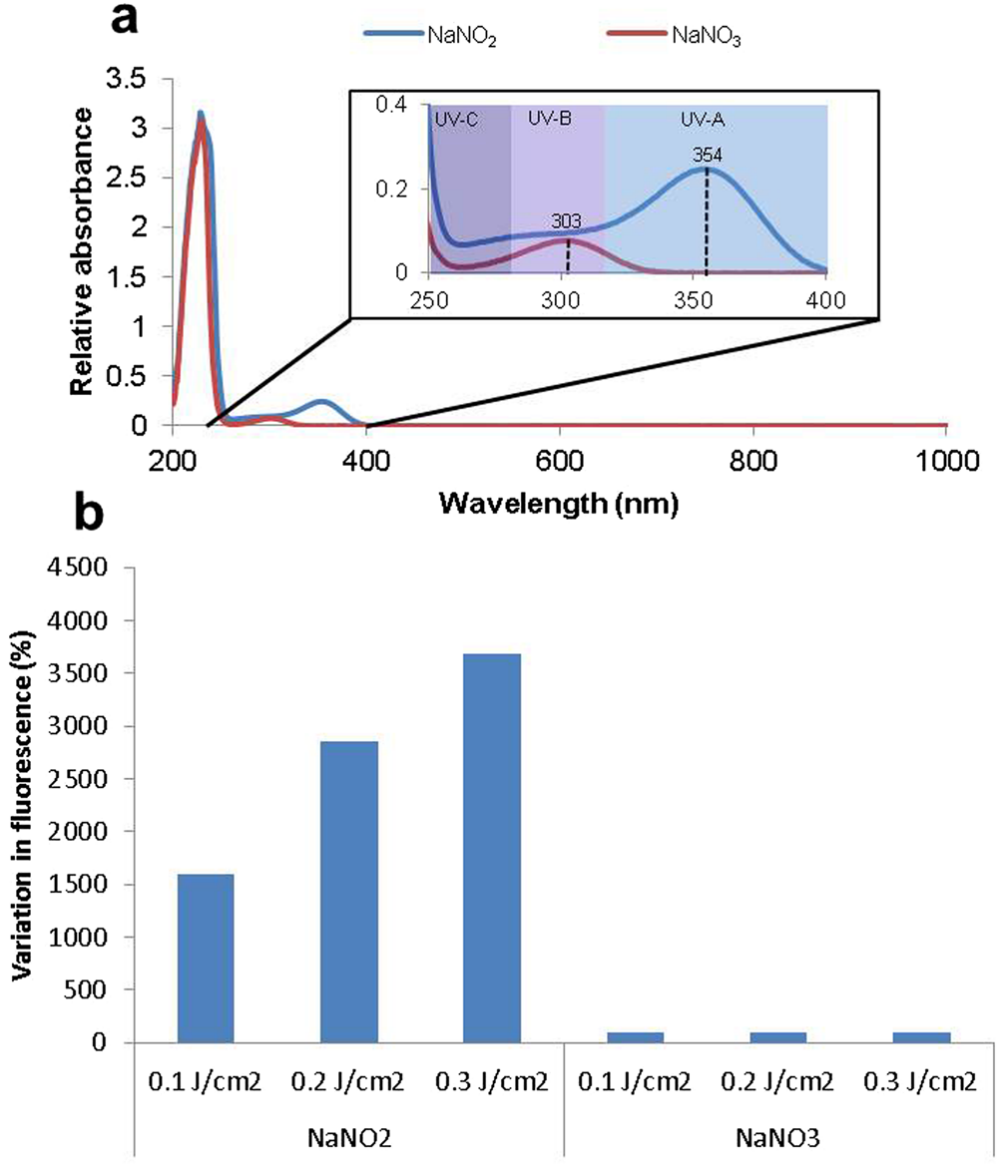
Comparison of sodium nitrite and nitrate in HBSS. (**a)** Solutions of 10 mM sodium nitrite and sodium nitrate in HBSS were assessed for absorption at wavelengths reaching from UV-C into the infra-red, with an inset expanded view of the UV region. (**b**) Solutions of 10 mM sodium nitrite and nitrate with increasing doses of UV-A in the presence of DAF-FM. Results are expressed as % change in fluorescence relative to unexposed control.

It was suggested that nitrite and nitrates might be potential “NO stores” that absorb energy from UV photons, which liberate NO^17^. To investigate the likelihood of this, UV-visible spectrophotometry of 10 mM sodium nitrite and 10 mM sodium nitrate solutions was performed (Fig. 6a). Apart from the high absorption in the UV-C region which is irrelevant to terrestrial solar-UV, sodium nitrite had an absorbance peak at ~354 nm, and nitrate at ~303 nm. The peak of nitrite absorbance is remarkably similar to the wavelengths we have demonstrated to induce NO production in cells. To ascertain whether the absorption of UV-A actually results in the production of NO, we irradiated sodium nitrite and nitrate and measured any NO that was released. When exposed to UV-A sodium nitrite solution released NO, as measured using DAF-FM, in a dose-dependent manner (Fig. 6b). However, sodium nitrate solution showed almost no NO production under the same conditions. These results strongly point to nitrites as the source of UV-A-induced NO production.

### Wavelength dependence of NO production

**Figure 7:**
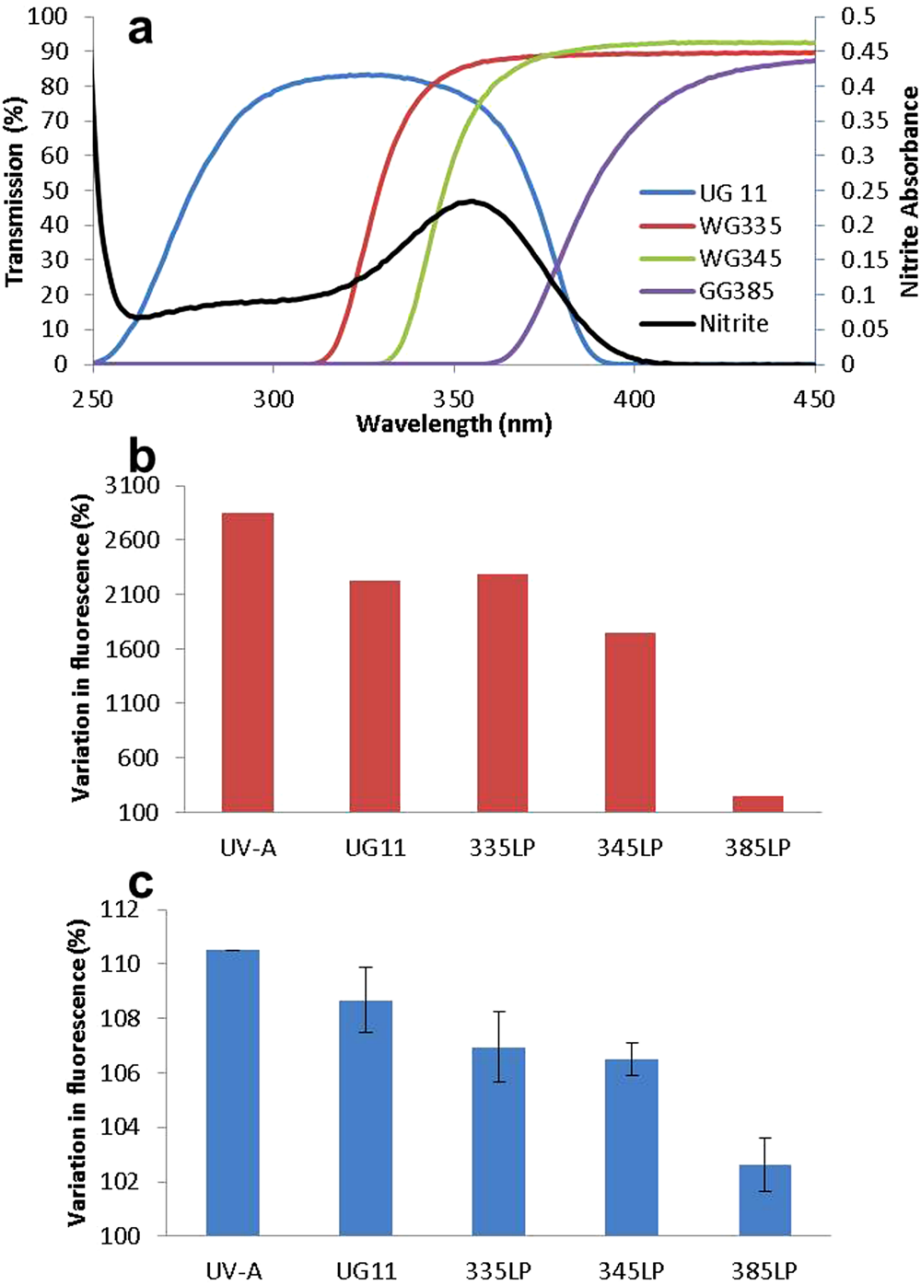
Wavelength-dependence of NO production in nitrite solution and FSKs. (**a**) The transmission profiles of 4 filters, one of which is a bandpass filter (UG11), which has two cut on/off points and the other three WG335, WG345 and GG385 are long-pass filters with single cut on/off points. The absorbance profile of NaNO_2_ is also shown (black line with right-hand y-axis) for reference. **(b**) NO production from aqueous nitrite solution reducing as the shorter UV-A wavelengths are progressively blocked by the use of UV-blocking filters. Results are expressed as % change in fluorescence relative to unexposed control. (**c**) A similar pattern is observed in FSKs which show NO production decreasing with the use of UV-blocking filters. Results are expressed as % change in fluorescence relative to unexposed control.

Having established nitrite’s responsiveness to UV-A, we used it to further investigate the wavelengths of light within UV-A that are able to trigger NO production. We used a set of UV filters to selectively block parts of the UV-A spectrum in a series of exposures. The filters used were a UV bandpass filter (UG11), which allows transmission of light with wavelengths between approximately 260 and 385 nm, and three long-pass filters; WG335, WG345 and GG385, with a 50% transmission cut-on wavelengths of approximately 329, 347 and 388 nm respectively. Figure 7a shows the UV transmission profiles obtained from these filters, super-imposed on the absorbance profile of sodium nitrite solution across UV-A. Firstly, the effect on NO production from aqueous sodium nitrite solution with UV-A irradiation was measured without or with these filters (Fig. 7b). With the UG11, WG335 and WG345 filters, a slight reduction is seen in the NO level compared to a no-filter control, but when the GG385 was used, cutting off wavelengths below ~370 nm, the NO production falls away dramatically. This demonstrates the major involvement of the ~340–370 nm region of UV-A in nitrite to NO conversion. To see if this effect was seen with cells, DAF-FM DA-labelled FSKs were used in place of nitrite solution in a similar manner. Remarkably, the decreased NO production observed with the use of filters is similar to that seen with nitrite solution (Fig. 7c), with the most marked reduction observed with the GG385 filter indicating a major role of 340–370 nm UV in driving NO production in keratinocytes. This very strongly suggests that forms of nitrite are indeed the cellular stores from which NO is liberated in response to UV-A. It also demonstrates that it is the less energetic middle to longer wavelengths of UV-A ( > 340 nm) that are the major players involved in this process.

## Discussion

Keratinocytes make up approximately 90% of the cells in the epidermis^23^. They are the closest viable cells to the surface of the skin, and assumed to be exposed to the highest levels of both UV-A and UV-B. Results from past studies that used various cell lines of diverse origins have reported UV-B to be about a hundred times more destructive than UV-A^32, 33^. To ascertain the relative effects of these two UV radiations on normal human skin cells that we employed in our study, the dose of UV-B was appropriately reduced by two orders of magnitude so as to avoid inaccuracies that inevitably come to fore when vastly different quantities are measured within an assay. In spite of UV-B dose being a hundred times less than that of UV-A, the former inflicted visibly more double-strand breaks and about a hundred times more thymidine dimers. On a rough approximation per Joule of energy, UV-A caused 250 times fewer double-strand breaks and 15,000 times fewer thymidine dimers than UV-B. While this does not mean that UV-A is entirely benign, it does provide an important perspective on how much less damaging UV-A is to cells. This is of particular relevance in regards to health protection advice and potential use of UV-A in modulating circulatory conditions.

UV-A penetrates well into the dermal layer where other cell types reside; primarily fibroblasts and microvascular endothelial cells. Although our work (Fig. 2) and that of others show that keratinocytes respond to UV radiation by releasing NO, it was not clear if other dermal cell types that are within the penetration depth of UV-A are similarly responsive to UV-A. Our results reveal that in addition to keratinocytes, dermal microvascular endothelial cells are also very responsive to UV-A. This appears not to have been observed by Liu *et al*.^5^ where skin sections were stained for NO production following UV-A. It is easy to imagine that responsiveness of microvascular endothelial cells might have escaped notice in the context of a skin cross-section, where the overwhelming quantity of keratinocytes, which are very responsive to UV-A, are readily displayed, while blood vessels are represented by the cross-section of only a few cells. Regardless of the reason, the responsiveness of dermal microvascular endothelial cells to UV-A is manifestly clear as it was seen in isogenic cell-type comparison experiments (Fig. 3) and repeatedly seen in microvascular endothelial cells from all tested donors (Fig. 4).

This is a very relevant finding because to affect blood pressure, NO, or its daughter molecules, need to access the smooth muscle cells surrounding blood vessels, and perhaps also into the vascular network. If keratinocytes were the only skin cells that released NO in response to UV-A, as was hitherto assumed, the NO produced would have to contend with its fleeting existence (as it is a highly reactive compound) and the considerable distance between the epidermis and the blood vessels in the dermal layer. It seems unlikely that NO produced in keratinocytes, in the epidermal layer can successfully migrate to smooth muscle cells around a blood vessel, in the dermal layer without becoming oxidised to nitrite, or otherwise reacting with other molecules. Although, given the large number of keratinocytes in the skin, and that NO is a potent vasodilator, a small percentage of NO from the epidermis reaching the vessels in the dermis may be sufficient to elicit a tangible effect. Another alternative is the above mentioned possibility of secondary molecules, such as nitrites, being able to affect a biological effect.

Hence our observation that microvascular endothelial cells are able to produce NO in response to UV-A irradiation is particularly relevant due to their location within blood vessels, and the proximity of smooth muscle cells that control their diameter. Given that UV-A is able to penetrate to depths in the skin where it will be able to directly interact with endothelial cells lining blood vessels^8, 34, 35^, our finding that FSECs can produce NO in response to UV-A is worthy of emphasis. This could allow release of NO to the surrounding smooth muscle cells, causing an immediate dilatory effect. In addition to this, NO or its secondary products can be released directly into the blood stream, to be transported further away to have wider-ranging effects. This does not in any way reduce the importance of UV-induced NO produced by keratinocytes. Instead it ushers in the notion of multi-phase UV-A-induced dilatory effect on blood vessels. The immediate-early phase would be that which is mediated by NO released from microvascular endothelial cells and followed by the early phase which is mediated by NO secondary products that are derived from UV-A irradiated keratinocytes, and the late phase which is NOS-mediated, as previously reported^17, 25^. The studies using the solar simulator for irradiation, demonstrate that sunlight may be more efficacious than UV-A alone at producing NO in FSECs. This might be due to other wavelengths, including UV-B, acting upon nitrates and other molecules present, although this is speculation and requires further study to fully understand the processes occurring. However, the important point here is that including UV-B at environmentally relevant levels does not seem to block or inhibit the UV-A-driven production of NO.

Since FSKs and FSECs respond to UV-A with broadly similar sensitivities, keratinocytes were more often used in subsequent experiments due to the vastly greater extractable quantities from skin samples. It is encouraging that on average, keratinocytes and microvascular endothelial cells both increase NO production by approximately 7%, which may appear modest at first sight, but when we consider that human exposure to environmental sunlight would involve a significantly greater surface area and hence number of cells, these increases becomes more meaningful. In their paper, Liu *et al*.^5^ reported that even a modest change in diastolic blood pressure of 10 mmHg can result in a 2-fold reduction in cardio- and cerebro-vascular mortality risk^5, 36^. Of important consideration is that while vasodilation is positive in terms of reducing blood pressure, unfettered reduction of blood pressure would be highly undesirable. It would be sensible to assume that evolutionary processes would have honed the level of NO production by sunlight to be manageable for the organism as to avoid a detrimental or even lethal drop in blood pressure when moving from shade to full sun. In our studies we are unable to gauge the effect that a 7% variation in NO level may have *in vivo*, however this is something that clinical studies may be able to investigate and clarify.

The apparent universality of cells (keratinocytes, microvascular endothelial cell, fibroblasts and melanocytes) within the skin to produce NO, albeit at different levels, is consistent from an adaptation point of view, as possessing a large number of cells spread widely across the skin able to gently produce a bioactive molecule in response to environmental stimulus would seem more manageable than a small number of cells able to produce vast amounts of the same molecule.

The observed rise of NO following UV-A irradiation in adult keratinocytes is encouraging in two ways. Firstly it demonstrates that the responsiveness to UV-A is not a feature that is confined to young skin or either gender. Secondly, given that elevated blood pressure is a condition that is prevalent in the adult population, the maintained UV-responsiveness of skin cells into adulthood is highly encouraging in terms of potential interventions.

A consistent rise in the level of NO following UV-A irradiation was observed across the 22 FSK donors, albeit with variation in the magnitude of this increase. This consistent rise is encouraging as it suggests that if the increased level of NO seen is able to have a beneficial outcome such as lowering blood pressure, then the benefit may be enjoyed by many. While the increase in NO level is broadly similar between donors, the basal level of NO in the unexposed cells shows greater variability; there is an approximate 3.5-fold difference between the highest and lowest DAF-FM fluorescence signal values recorded. This is consistent with other studies that have found variation between the concentrations of NO-related products with *in vivo* skin studies^17^. The reason for the different basal levels of NO can only be speculated as deriving from genetic or epigenetic differences that determine NOS enzyme expression and activity in the different donors, affecting how they process identical quantities of NO precursors, which is presumed to be primarily arginine, from their culture media.

Interestingly, the rise in NO observed following UV-A irradiation of the 22 donor cells seemed unrelated to the basal NO level. This is consistent with the interpretation that while basal NO level is determined primarily by NOS activity within the cells of the individual donors, the UV-A-induced surge of NO is drawn from NO stores in the cells, and hence could be less prone to genetic variability of NOS activity. As nitrite levels are established and maintained through dietary intake, especially of vegetables, it is possible that *in vivo* UV-A-induced NO production might still be affected by inter-individual differences, through diet, life-style and environmental as well as genetic influences.

In regards to “NO stores”, the chemistry of NO production would allow for nitrite and nitrate to act as potential NO stores in cells. The absorption peaks (Fig. 7) demonstrate very clearly that nitrite absorbs radiation at wavelengths that are well within the UV-A spectrum. Utilising filters to vary the wavelengths used to induce NO production from nitrite solution produced results that are remarkably similar to the same filtered wavelengths used with NO production in FSKs. These observations lend very strong support for nitrite being the primary reservoir for UV-A-induced NO production.

In spite of the clear rise in NO levels in UV-A-irradiated FSKs and FSECs, there is a question of which cells in the skin have the greatest stores of UV-A-convertible nitrites. The large number of keratinocytes in the skin may allow low concentrations of nitrite held in individual cells to translate into large stores when considering the skin *en masse* as an organ. Endothelial cells are involved in endogenous control of blood pressure, through the release of NOS-derived NO when required, and so are another possible source of photochemically-mediated NO generation.

In these cell studies the response to a single dose of UV-A was assessed. Obvious questions present themselves with regards to the response to chronic exposures, dose responsiveness, a threshold for effect, cumulative effects of repeated exposures, refractory periods, and clinical relevance, among others. If the effect is sustained following repeated exposures this could provide an important dimension to be considered when formulating sun exposure advice for the public in general, as well as targeting more specific advice to those with hypertension.

While modern sun protection approaches have moved towards blocking much of the UV spectrum to try to prevent the negative effects of UV, more discerning protection methods could be envisaged which block the most harmful wavelengths, while allowing less harmful and more beneficial ones through. As artificial UV for medical treatment is already used for conditions such as psoriasis, the feasibility of using carefully defined UV sources, in combination with evidence-based advice and careful monitoring, for medical benefit has potential. This could be especially relevant in the winter months when sunlight is in short supply.

There is debate currently ongoing regarding the balance of advice between exposure to UV-B wavelengths to allow vitamin D generation and avoidance of these wavelengths due to the direct DNA damage they cause^37–39^. A major difficulty is due to the fact that vitamin D production is stimulated by UV wavelengths that are also highly damaging to DNA. It is fortuitous that this challenge is vastly reduced with regards to the likely blood pressure-lowering effects of UV because of the separation of biological effects (vasodilation and DNA damage) between UV-A and UV-B. We would be remiss not to consider and promote the damaging effects of UV radiation, and that UV-A is still capable of causing some damage to DNA, but the magnitude of this is indeed very small compared to that caused by UV-B. With a greater appreciation of the positive as well as negative effects of different UV wavelengths, a better understanding of ‘essential’, ‘desirable’ and ‘dangerous’ wavebands can only serve to improve advice, and possibly encourage the formulation of sun protective creams and clothing that reduce the detrimental effects of sunlight while allowing the beneficial ones to be enjoyed, as well as promoting attitudes and behaviours leading to the idea of ‘healthy sun exposure’.

## Materials and Methods

### Cells

Foreskin keratinocytes (FSK), melanocytes (FSM), microvascular endothelial cells (FSEC) and fibroblasts (FSF) were isolated from foreskins collected from neonatal donors (approximately 5–7 days old) from routine circumcision. Adult keratinocytes were obtained from samples of adult tissue, following the same process as for FSKs. Obtainment of informed content preceding collection of foreskins was conducted under the approval of the Oxford Research Ethics Committee; reference 10/H0605/1. Following resection, the tissue was placed in transport medium (DMEM supplemented with 10% FBS (Gibco, 10500064), penicillin, streptomycin, amphotericin and gentamycin) and transported to the laboratory at ambient temperature. The tissue was then aseptically cut into oblongs of 5–7 mm each side and digested overnight at 4 °C with 0.5 mg/ml Liberase DH in CnT-07 keratinocyte medium (CellnTech). Following digestion, the epidermis was peeled from the dermal layer, transferred to trypsin-EDTA, and the tissue mechanically dissociated to form a single-cell suspension. After pelleting, the cells were resuspended in either keratinocyte CnT-07 or melanocyte MGM M2 media (PromoCell). Cells were then seeded into collagen and fibronectin-coated or gelatin-coated vessels to select for FSK or FSM respectively.

To isolate FSFs, dermal pieces were grown in DMEM supplemented with 10% FBS and grown as explants. The remaining dermal tissue was digested in 2.5 mg/ml collagenase in HBSS (with Ca and Mg) at 37 °C with frequent agitation for 1 h, passed through a 70 μm cell strainer and selected using CD31 magnetic Dynabead positive selection (Life Technologies, 11155D). Selected cells were then seeded on a gelatin-coated flask in Endothelial Cell Growth Medium MV (PromoCell, C-22020).

All cells were maintained in a 37 °C, 5% CO_2_ humidified environment.

### UV Exposure

UV exposures were performed using a BIO-SUN UV exposure system (Vilber Lourmat). This device has four 30 W UV-A tubes with an output peak at 365 nm, and two 30 W UV-B tubes with an output peak at 312 nm. Output and spectra were analysed with a calibrated QE65000 spectral radiometer (Ocean Optics) coupled with a 600 µm optical fibre to a D7H cosine diffuser (Bentham Instruments).

For these studies doses of 3, 6, 9 and 12 J/cm^2^ UV-A and 0.03, 0.06, 0.09 and 0.12 J/cm^2^ UV-B were used as required. The BIO-SUN incorporates real-time irradiance monitoring, and this was used to ensure consistent dose delivery.

The solar simulator used was a Sol 2-R unit (Honle), and the output spectrum was analysed using an Exemplar + spectral radiaometer (B&W Tek).

Spectra of both exposure setups are given in Supplementary Figure 2.

### Western blotting

FSKs were grown in 10 cm cell culture dishes. Media was removed, and the cells rinsed with phosphate buffered saline (PBS). Six millilitres of pre-warmed PBS was added to the cells and exposure to 9 J/cm^2^ UV-A and/or 0.09 J/cm^2^ UV-B was conducted. Exposures were performed in duplicate, with one set of plates harvested by scraping immediately, the other was replenished with fresh media and returned to the incubator for 5 hours, before being scraped into PBS. Protein was extracted from cell pellets by resuspending in 100 µl PBS and adding 100 µl 2X SDS Protein Lysis Buffer (2% SDS in 0.1 M Tris pH 8.0). The lysate was passed through a QIAShredder Column (Qiagen), and protein concentration was measured using a Pierce BCA Protein Assay Kit (Thermo Scientific 23225). Ten micrograms of protein per sample lane was loaded into a 4–20% gradient gel (Bio-Rad), and run at 120 V for 1 hour. Transfer to PVFD membrane was performed with a Trans Blot Turbo unit (BioRad). The membrane was blocked with 10% milk powder in Tris-Buffered Saline (TBST) for one hour at room temperature. Primary antibodies were incubated overnight at room temperature in TBST. Anti-GAPDH (Santa Cruz sc-25778) was used at a dilution of 1:1,000 and anti-γ-H2AX (Cell Signalling 9718 S) was used at a dilution of 1:5,000. Secondary antibody HRP-conjugated anti-rabbit was used at a dilution of 1:10,000, and incubated for one hour at room temperature.

### ELISA for 6–4PP

To measure the concentration of 6–4PP induced following UV-irradiation, an OxiSelect UV-induced DNA Damage ELISA Kit (Cell Biolabs) was employed. DNA was extracted from irradiated cells using a DNeasy Blood and Tissue Kit (Qiagen). The ELISA was performed following the manufacturer’s protocol.

### Nitric oxide detection using DAF-FM DA

The use of DAF-FM DA in detecting NO has been reported as a specific and suitable chemical^40^. The method of detection used here was inspired by a method previously reported by Paul *et al*.^41^.

DAF-FM DA loading was performed by adding 1 µl of 5 mM stock of DAF-FM DA (Molecular Probes D23844) in DMSO per 1 ml of culture media, giving a final concentration of 5 µM, and incubating for 45 minutes at 37 °C. The media containing DAF-FM DA was then removed, and the cells washed with PBS and removed from the plate by the action of Trypsin-EDTA solution. Cells were then centrifuged, and resuspended in PBS. The cell suspensions were then transferred to 12-well plates for UV exposure.

Following UV exposure, 150 µl samples of the cell suspensions were loaded into 96-well plates, had 150 µl PBS with 50 µg/ml propidium iodide (PI) (Sigma) added, and the plates read with a Guava EasyCyte HT flow cytometer (Merk Millipore). Cells positive for PI were excluded from the study, and the average DAF-FM FL1 signal for intact cells was compared. For the 22 donor FSK study the experiment was run twice, with two separate sets of cultures and exposures.

### Data Availability

The datasets generated during and/or analysed during the current study are available from the corresponding author on reasonable request.

## Electronic supplementary material

Supplementary Figures
