# Supplementary material for: Ultraviolet Radiation-Induced Production of Nitric Oxide:A multi-cell and multi-donor analysis
Source: Sci Rep. 2017 Sep 11;7:11105. doi: 10.1038/s41598-017-11567-5 (PMC5593895; doi:10.1038/s41598-017-11567-5)

Ultraviolet Radiation-Induced Production of Nitric Oxide: A multi-cell and multi-donor analysis

Graham Holliman[1]*, Donna Lowe[1], Howard Cohen[2], Sarah Felton[3] and Ken Raj[1]

[1] Radiation Effects Department, Centre for Radiation, Chemical and Environmental Hazards, Public Health England (PHE), Chilton, Oxfordshire, OX11 0RQ United Kingdom
[2] Elizabeth House, 515 Limpsfield Road, Warlingham, Surrey, CR6 9LF
[3] Oxford University Hospitals NHS Foundation Trust, Old Road, Oxford, OX3 7LJ

* Corresponding Author
Graham.Holliman@phe.gov.uk
CRCE, PHE,
Chilton,
Oxfordshire,
OX11 0RQ
Tel: 01235 825125

Short Title : UV-A and NO in human skin cells

Supplementary Figure 1

Complete scans of western blot membranes that were cropped for inclusion in Figure 1a

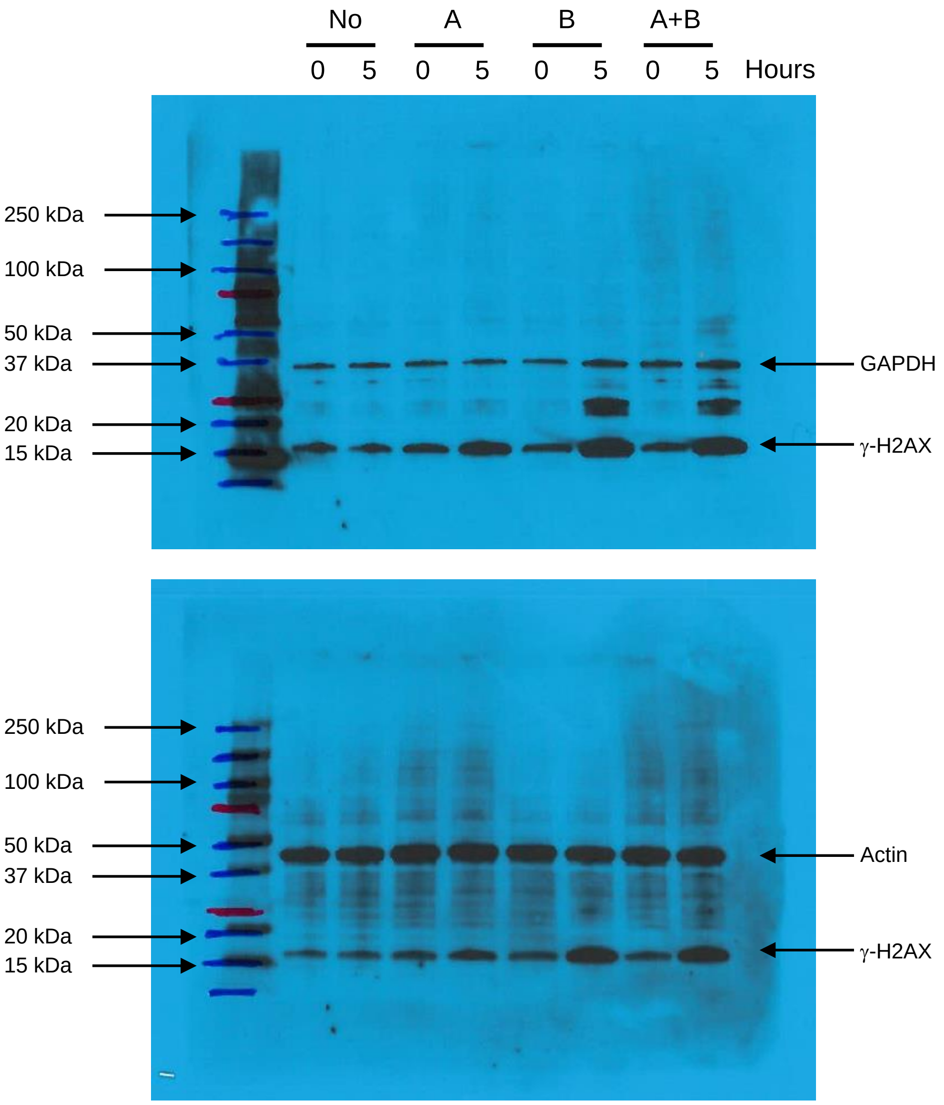

Supplementary Figure 2

Output spectra of BIO-SUN system and Honle solar simulator.

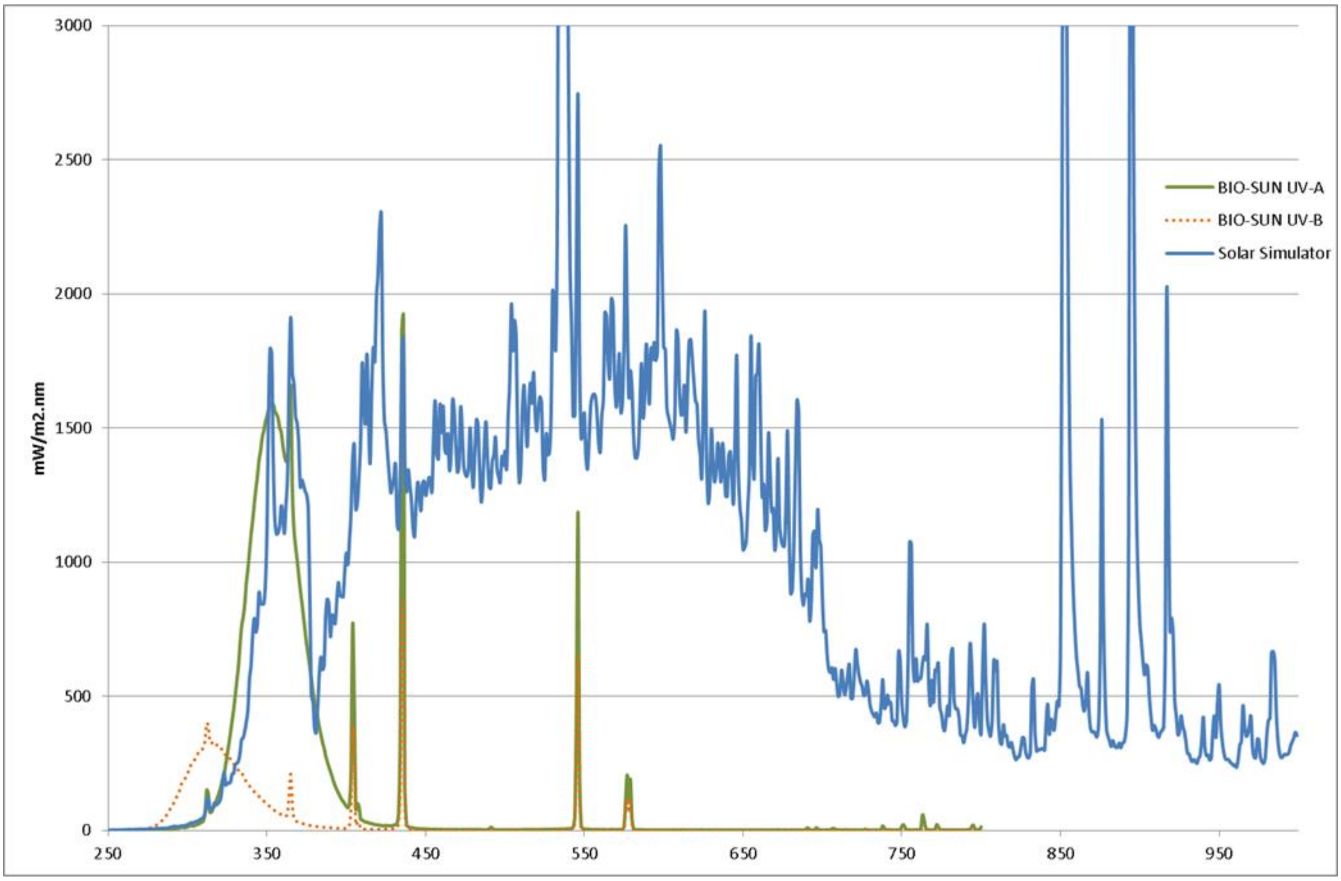

Supplement: Supplementary file 1 — Supplementary Figures [file 41598_2017_11567_MOESM1_ESM.pdf]
